# Bacterioplankton Activity in a Meso-eutrophic Subtropical Coastal Lagoon

**DOI:** 10.1155/2018/3209605

**Published:** 2018-10-03

**Authors:** Maria L. Schmitz Fontes, Heloísa Fernandes, Manoela Brandão, Mariana Coutinho Hennemann, Raquel Aparecida Loss, Valdelúcia Maria Alves de Souza Grinevicius, Denise Tonetta, Karina Cesca, Mônica Hessel Silveira, Mara Bedin, Derce Recouvreux, Regina Vasconcellos Antônio

**Affiliations:** ^1^Departamento de Ecologia e Zoologia, Centro de Ciências Biológicas, Universidade Federal de Santa Catarina, Trindade, 88040-970 Florianópolis, SC, Brazil; ^2^PPGOCEANO, Departamento de Geociências, Centro de Filosofia e Ciências Humanas, Universidade Federal de Santa Catarina, Trindade, 88040-970 Florianópolis, SC, Brazil; ^3^Departamento de Engenharia Sanitária, Centro Tecnológico, Universidade Federal de Santa Catarina, Trindade, 88040-970 Florianópolis, SC, Brazil; ^4^Departamento de Engenharia Química e Engenharia de Alimentos, Centro Tecnológico, Universidade Federal de Santa Catarina, Trindade, 88040-970 Florianópolis, SC, Brazil; ^5^Departamento deBioquímica, Centro de Ciências Biológicas, Universidade Federal de Santa Catarina, Trindade, 88040-970 Florianópolis, SC, Brazil; ^6^Campus Araranguá, Universidade Federal de Santa Catarina, Trindade, 88000-000 Araranguá, SC, Brazil

## Abstract

The aim of this study was to investigate whether the bacterioplankton activity in the meso-eutrophic Conceição Lagoon would increase significantly under allochthonous inputs of inorganic nutrients and organic carbon. Abundance and biomass of bacterioplankton were evaluated under three treatments: light (14 h light/10 h dark), complete darkness (dark-control), and nutrient (C + N + P—dark, 100 : 10 : 1) enrichments during 72 h. Nutrient enrichments promoted a significant increase in abundance (maximum of 19.0 ×10^9^ cells·L^−1^ in the first 32 hours) and biomass of the heterotrophic bacterioplankton, which induced the formation of large clusters. Bacterial biomass remained constant in the non-enriched incubations (dark-control and light). Bacterial growth rates were significantly higher after nutrient additions (1.35 d^−1^), followed by control (0.79 d^−1^), and light (0.63 d^−1^) treatments, which were statistically equal (*p* > 0.05). Bacterial production rates were also significantly higher under nutrient additions (1.28 d^−1^), compared to the control and light (0.50 d^−1^ and 0.44 d^−1^, respectively), demonstrating that bacterial growth and production in this meso-eutrophic lagoon are under an immediate “bottom-up” regulation, followed by a potential top-down effect. These facts reinforce the urgency on improving the local wastewater management plan in order to prevent further expansion of anoxic waters.

## 1. Introduction

The comprehension of the pelagic bacterioplankton abundance and activity in coastal aquatic environments is of major importance in order to predict shifts in the microbial communities in response to frequent changes in such systems [1]. The inputs of organic material and inorganic nutrients to coastal lagoons mainly from watershed runoffs, infiltration, precipitation, air and sea exchanges can have a direct influence on activity and composition of microbial communities and, consequently, in the ecosystem function [2, 3]. Coastal lagoons are located in the transitional region between terrestrial and marine environments, and are considered highly dynamic environments, subjected to abrupt changes in physicochemical variables, residence times, and recipients of multiple sources of nutrients [3, 4].

Heterotrophic bacteria play important roles in the remineralization and transformation processes between dissolved organic carbon (DOC) and particulate organic carbon (POC) [5, 6], which will then be available to higher trophic levels through the microbial loop [7]. The functionality of bacterioplankton, predominantly net sink or link of carbon in a system, will depend on the chemical composition of the DOC pool [8], if predation is absent or minimized.

However, the cause-effect relationship of labile carbon forms and/or inorganic nutrients on the bacterial activity is not clear yet. Some studies have shown a positive effect of dissolved inorganic nitrogen and phosphate enrichment on marine bacterial growth [9–12], while others demonstrated no effect [13]. With the simultaneous addition of inorganic nutrients and glucose, several studies have shown a significant response in bacterial abundance and production [14–17]. The heterogeneity observed in the results has been related to the different trophic states of each environment [9].

In coastal ecosystems such as the Conceição Lagoon, a meso-eutrophic coastal lagoon [18], additional inputs of allochthonous material can stimulate heterotrophic microorganisms and consequently lead to oxygen depletion [19–21]. Anoxia occurrence has increased significantly in the last 15 years in the central area of the lagoon [18], and its trophic state has changed from oligo-mesotrophic to meso-eutrophic in the same period of time [18]. Thus, we hypothesized that bacterioplankton activity, determined here by bacterial production and growth, would increase in this meso-eutrophic system if exposed to simultaneous external inorganic nutrients' sources and organic matter.

## 2. Materials and Methods

### 2.1. Water Sampling and Study Site

**Figure 1:**
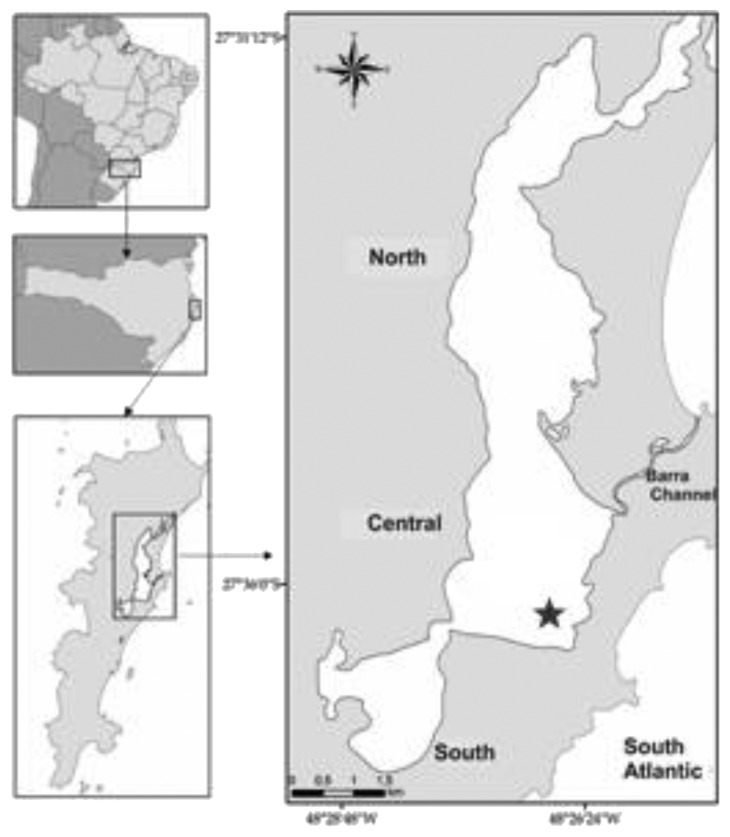
Location of the Conceição Lagoon (grey), Santa Catarina Island, Florianópolis, Brazil. Sampling site is represented by a star symbol (adapted from Fontes and Abreu [19]).

A 20 L water sample was collected at subsurface in the central sector of Conceição Lagoon on the 2nd of November, 2011, and was kept at low temperature until transported to the laboratory. The lagoon is located between 27°30′17″ and 27°37′36″S and 48°25′30″ and 48°29′54″W (Figure 1).

Conceição Lagoon is connected to the Atlantic Ocean by a 2 km long channel, which drives a salinity variability in the central sector from 17.6 up to 30 [19, 22]. Water temperature during the experiment was maintained 24°C; usual dissolved inorganic nitrogen (ammonium + nitrate + nitrite) and dissolved inorganic phosphorus (phosphate) concentrations range between 1.0 and 13.2 *µ*M and between 0.09 and 0.52 *µ*M, respectively; with average DIN : DIP ratios above 25 : 1 [22]. Chlorophyll a concentrations average between 3.5 and 4.0 *µ*g·L^−1^ [19, 20, 22]. Additionally, continuous runoffs rich in untreated (raw) domestic sewage flow directly into its water body [22, 23]. The average domestic sewage volume discharged into the system has been calculated to be around 160 m^3^ of sewage month^−1^, corresponding to 300 kg (biological oxygen demand, BOD_5_ day)^−1^ [24]. Due to this elevated sewage input, a considerable increase in dissolved inorganic nitrogen (ammonium, nitrite, and nitrate) concentrations has been observed in the lagoon water since 1980s [25].

### 2.2. Culture Preparation and Experimental Procedure

Water aliquots were collected from Conceição Lagoon and carefully filtered through a cellulose acetate membrane (Millipore 47 mm in diameter and 0.2 *µ*m in pore size) in the laboratory. One liter of the filtered water or medium (dilution of the sample resulted in a reduction in the amount of grazers [14]) was transferred onto each of three sterile glass recipients resulting in 70% of filtered medium and 30% of inoculum (whole water—no previous filtration), equivalent to a volume of 1.43 L per replicate. Bacterioplankton cultures received the following labels according to the treatment: (1) light (*in situ* light intensity and photoperiod: 14 h light, 10 h dark, room temperature); (2) control (dark, room temperature); and (3) nutrients (C + N + P = glucose, NH_4_Cl, and K_2_HPO_4_ additions at final concentrations of 100 *µ*M C : 10 *µ*M N : 1 *µ*M P, dark, room temperature). All treatments were kept under the described conditions for 72 h. The reason why control and nutrients' treatments were kept in the dark was to prevent the accumulation of autotrophic biomass (presence of photoautotrophic bacteria and picoeukaryotes) throughout the experiment, which would interfere with the results by competition or release of extracellular DOC. On the other hand, light treatment was important for the effects of light/dark cycles on bacterial activity when compared to complete darkness.

### 2.3. Bacterial Counts and Biomass

Aliquots of 15 ml were taken from each treatment at 12 h intervals and fixed with PFA (paraformaldehyde) at 2% (final concentration), making 7 estimates for each treatment (*t*_0_, *t*_12_, *t*_24_, *t*_36_, *t*_48_, *t*_60_, and *t*_72_). The aliquots were used to determine bacterial abundance, biomass, and size or biovolume. One ml of aliquots from each treatment was filtered through 0.2 *µ*m dark polycarbonate membranes (diameter of 25 mm) (Millipore) and stained with 4′,6-diamidino-2-phenylindole (DAPI) for 15 min (1 *µ*g·ml^−1^, final concentration).

Filters were mounted onto slides, and bacterial cells were counted using an epifluorescence microscope (Olympus BX-40) [26]. From each filter, ten random fields were counted and at least 200 bacterial cells were measured. Length, width, elongation, and area of each cell were measured using the freeware “UTHSCSA Image Tool” (University of Texas Health Science Center, San Antonio, Texas, US) [27]. Bacterial biovolume was calculated from length and width measurements and biomass as function of biovolume using the following algorithm: *B* = 120 *V*^0.72^, where *B* = biomass; *V* = biovolume (*μ*m^3^); 120 = conversion factor in fg·C·*μ*m^−3^ [28]. Total biomass was calculated by multiplying the average cellular biomass by bacterial abundance.

### 2.4. Data Analysis

Bacterial growth was calculated as the increase in cellular density from the beginning to the end of the exponential growth period. Bacterial production was calculated in the same manner using bacterial biomass data by the time. Bacterioplankton specific growth rates (*µ*) and biomass production rates were calculated as the slope of the linear regression of ln-transformed bacterial abundance or biomass with time during the exponential growth [29]. Daily growth rate (daily *µ*) and daily bacterial production rates were obtained by multiplying the hourly growth rate by 24. Doubling time was calculated by dividing 0.69 (ln 2) by the daily *µ*. Comparison between bacterial growth rates and production rates (linear regression angular coefficient) was made by analysis of covariance (ANCOVA), with subsequent multiple comparisons performed using Tukey's test with a statistical significance level of *p* ≤ 0.05. Density, biomass, and biovolume means were compared by analysis of variance (ANOVA) or *t*-test, if the case, of (log + 1)-transformed data, followed by Tukey's test (significance level of *p* ≤ 0.05) [30, 31]. Statistical tests were conducted using Statistica 7 software.

## 3. Results

**Figure 2:**
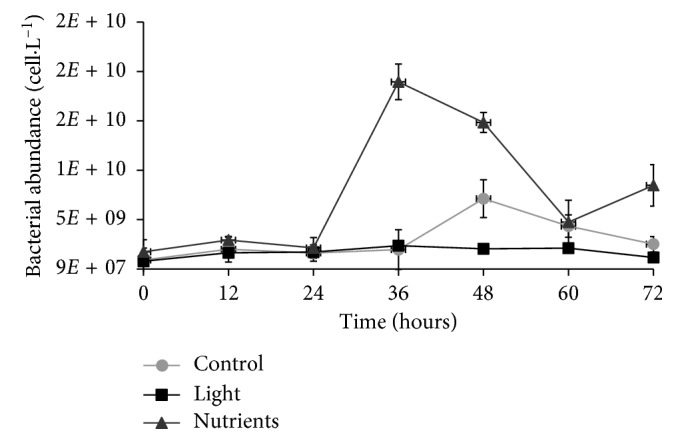
Bacterial abundance (mean ± standard error) in the three treatments during the 72 h incubation period. Treatments: dark-control = control, 14 h light/10 h dark cycle = light, and dark C + N + P enrichment = nutrients.

Samples showed different abundance and biomass values among treatments and time. The mean number of cells (cells·L^−1^) in each treatment after 72 h of incubation was higher in the nutrient treatment (7.44 × 10^9^ ± 2.55 × 10^9^ cells·L^−1^), followed by the dark-control (3.00 × 10^9^ ± 8.04 × 10^8^ cells·L^−1^) and light flasks (1.77 × 10^9^ ± 2.08 × 10^8^ cells·L^−1^). Bacterial abundance increased exponentially until 36 h (maximum 1.9 × 10^10^ cells·L^−1^), followed by a decrease and a second small increase between 60 and 72 h in the nutrient treatment. In the dark-control treatment, the number of cells increased until 48 h (maximum 7.18 × 10^9^ cells·L^−1^). In the light flasks, samples showed a lower abundance when compared to the other treatments and maintained approximately constant values throughout the experiment (Figure 2).

**Figure 3:**
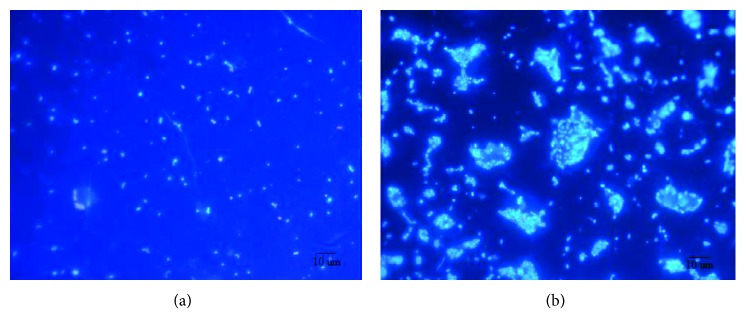
Epifluorescence microphotographs (1000x) of bacteria stained with DAPI at 12 h (a) and 72 h (b) of the experiments in the C + N + P treatment.

**Figure 4:**
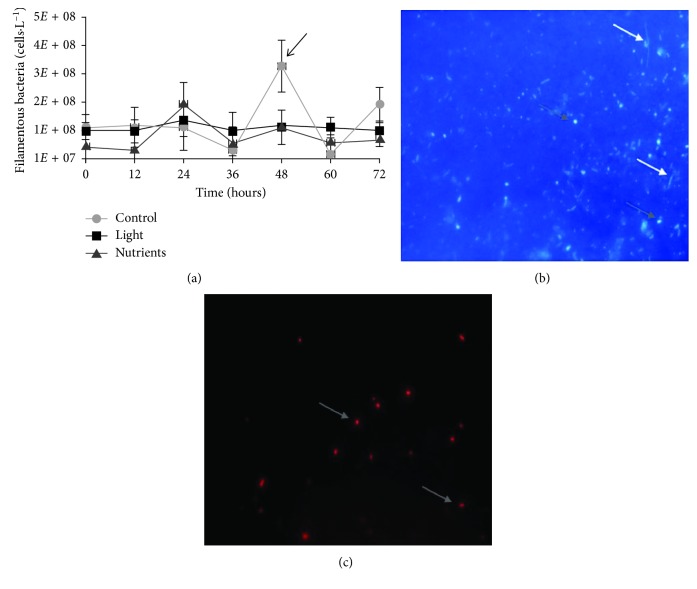
Filamentous bacterial abundance (mean ± standard error) (a) in the three treatments during the 72 h incubation period. Treatments: dark-control = control, 14 h light/10 h dark cycle = light, and dark C + N + P enrichment = nutrients. Examples of filamentous bacteria (b) and cyanobacteria (c) present in the dark-control treatment at the 48 h. (b) DAPI-stained cells, heterotrophic filamentous bacteria (indicated by white arrows). (c) Autofluorescence of cyanobacteria in red (indicated by grey arrows).

Considering cells arrangement in each treatment, there was an increase in the occurrence of cells clustering and size after 36 h of incubation only in the nutrient enrichment, with up to 200 cells clustered together (Figure 3). In addition, the abundance of filamentous bacteria increased between 12 and 24 h in the nutrients treatment, while in the dark-control, a smaller and delayed increase occurred between 36 and 48 h. In the light treatment, constant numbers of filamentous bacteria were observed throughout the experiment (Figure 4). However, no significant differences were observed concerning filamentous bacterial abundance among treatments (one-way ANOVA, *p* > 0.05). Considering the relative abundance of filamentous bacteria, a stronger contribution of this portion of the community was detected in the light and dark-control flasks (5.6% and 7.1%, respectively), against 2.5% contribution in the ones that received nutrients.

**Figure 5:**
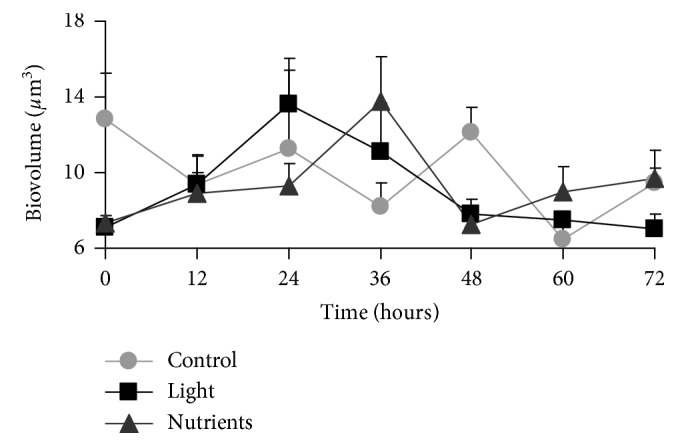
Cellular biovolume in *µ*m^3^ (mean ± standard error) in the three treatments during the 72 h incubation period. Treatments: dark-control = control, 14 h light/10 h dark cycle = light, and dark C + N + P enrichment = nutrients.

**Figure 6:**
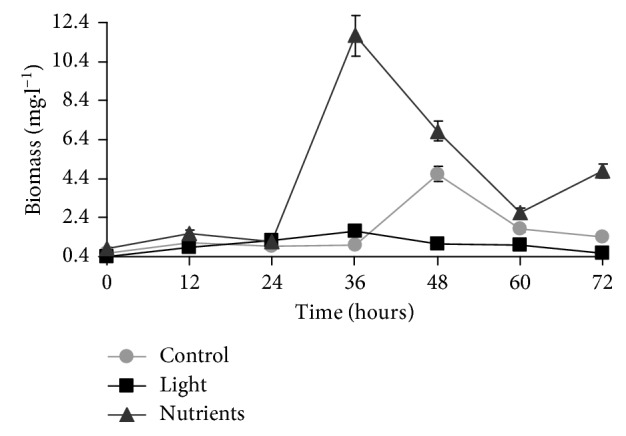
Bacterial biomass (mg·C·L^−1^ + standard error) in the three treatments during the 72 h incubation period. Treatments: dark-control = control, 14 h light/10 h dark cycle = light, and dark C + N + P enrichment = nutrients.

The bacterial size estimated as cellular biovolume (*µ*m^3^) has been used to calculate cellular biomass in function of C availability (*µ*g·C·cell^−1^). As a result, mean cellular biovolume ranged between 6.45 and 13.65 *µ*m^3^, and biomass, as a consequence, between 417.82 and 678.36 *µ*g·C·cell^−1^. The highest averages of biovolume were observed in the nutrients treatment at 36 h (13.65 *µ*m^3^) and in the light at 24 h (13.58 *µ*m^3^) (Figure 5). High oscillations in the mean biovolume can be attributed to different contributions of filamentous bacteria and to the presence of different bacteria morphotypes. However, biovolume was not significantly different among treatments (one-way ANOVA, *p* > 0.05) (Table 1). On the other hand, considering total biomass (carbon per volume unit (mg·C·L^−1^)), the highest biomass was registered in the nutrients treatment, especially between 24 and 36 h (with a peak of 12.3 mg·C·L^−1^) (Figure 6), similarly to what was shown for bacterial abundance (Figure 2).

Bacterial abundance and biomass were higher under nutrients additions when compared to the dark-control and light treatments (one-way ANOVA, *p* < 0.05) (Table 1). Bacterial growth rates were 0.79 d^−1^, 1.34 d^−1^, and 0.63 d^−1^ in the dark-control, nutrients, and light treatments, respectively. Bacterial production rates oscillated from 0.44 to 1.13 d^−1^ (Table 2). The ANCOVA result showed that bacterial growth and biomass production rates differed among treatments (ANCOVA: *F*_0.05;(1);2;9_ = 9.00 for growth and ANCOVA: *F*_0.05;(1);2;9_ = 5.08 for bacterial production). The difference among slopes was tested, and it was observed that bacterial growth and production were significantly higher in the nutrients treatment, while growth and production were similar in light and dark-control incubations (Table 2).

Doubling times (0.51 d^−1^, 0.87 d^−1^, and 1.10 d^−1^ for nutrients, dark-control, and light, respectively) showed that the heterotrophic bacterial community went through two duplications per day in the nutrients treatment, while in natural conditions (dark-control treatment), bacterioplankton duplicated once a day (Table 2).

When changes in cellular biovolume and carbon biomass are considered, slightly different results were observed, since both nutrients and light treatments showed small increases in the parameters at the end of the 72 h incubation period.

## 4. Discussion

Bacterioplankton activity in this meso-eutrophic lagoon was significantly stimulated after the addition of dissolved organic carbon and inorganic nutrients, demonstrating the vulnerability of this system to external sources of organic and inorganic matter.

The average cellular size, 13 *µ*m^3^, did not vary significantly throughout the experiment; however, bacterial cells showed a great morphological variability. Naturally, isolated marine bacteria show low growth rates under environmental conditions [32]. These authors reported that cells with a biovolume of 0.008 *µ*m^3^ have growth rates of 0.3 d^−1^, while 0.3 *µ*m^3^ cells show growth rates of 1.4 d^−1^. The average biovolume found in our study was 13 *µ*m^3^, two orders of magnitude higher than usually found in marine environments. However, it is important to highlight the presence and the relative abundance of filamentous bacteria, especially around 48 h in the control treatment. The predominance of heterotrophic filamentous bacteria has already been described previously in the Conceição Lagoon as positively related to the coccoid cyanobacteria of genus *Synechococcus* [19], as shown in Figures 4(b) and 4(c). This has been described as a strategy to avoid predation, when bacteria alter their size towards filamentous forms when exposed to grazing pressure [33–35].

Some studies have suggested that viral lysis and predation by protists are the main regulators of bacterial growth and production, many times more important than resources availability [36–38]. However, in environments with N : P ratios above the Redfield ratio, where phosphorous is the limiting resource, as in Conceição Lagoon [22, 23], bottom-up control of bacterial growth and/or biomass has a much more important role than top-down control by bacterivory [39, 40]. Therefore, grazing cannot be ignored as a secondary regulator on the activity of bacterioplankton. The significant decrease of bacteria abundance between 36 and 60 h in the nutrients treatment was concurrent with a slight decrease in the average bacterial biovolume, which is illustrated by the formation of several clusters of bacterial cells resulting on smaller biovolume when attached to each other. There are a few hypotheses for this outcome: predation scape strategy, increase proximity, and signaling among cells to overcome resource limitation.

In Conceição Lagoon, bacterial growth rates and production doubled after the addition of nutrients, compared to the dark-control and light treatments. In a study carried out with samples from the oligotrophic Sargasso Sea, the additions of nitrogen, phosphorus, and carbon resulted in increasing bacterial production by 7 to 15 times when compared to control or inorganic nutrient additions alone [14]. This finding reinforces that bacterioplankton of oligotrophic environments (under constant starvation and competition pressure with phytoplankton) responds much faster after simultaneous additions of inorganic nutrients and carbon than those of meso-eutrophic environments.

Previous studies have shown contradicting results on the effect of nutrients and carbon enrichment on bacterial growth and secondary production (e.g., [40–46]). For example, a significant increase on bacterial production after the addition of nutrients and carbon are reported in a fjord in Norway [47], whereas no significant changes were described in the oligo-mesotrophic Uchiumi Bay, Japan [13]. The best explanation for such heterogeneity in growth and productivity of aquatic bacteria has been described to be the ecosystem's trophic state, as the primary response of bacteria to eutrophication is to increase their specific growth rates [48]. The ecosystem's trophic state is related to variables that directly or indirectly correlate with the system's primary production: total organic matter, dissolved oxygen concentration, total phosphorus, light penetration, and phytoplankton biomass indicated by chlorophyll a concentration [49, 50]. As Conceição Lagoon is under eutrophication, with persistent anoxic waters in the bottom of the central sector, the increase in aerobic respiration by microbes can be a major problem for the water quality of this ecosystem.

Thus, increasing nutrient loads (N and P) directly or indirectly (trophic state) will alter bacterial community composition by favouring certain bacterial populations that have the ability to rapidly consume these new available resources [51–55]. Our results show that bacterial activity remains unaltered when exposed to complete darkness (*t*-test - light x control - *p* > 0.05), and that under additions of inorganic nutrients and organic carbon, the total bacterial activity doubles in the first 24 hours. Apparently, there is an indicative of strong competition for nutrients in Conceição Lagoon.

The present study shows similar activities for total (auto + heterotrophic) bacterioplankton and strict heterotrophic bacteria when neither inorganic nutrient nor carbon is added onto the lagoon. Nevertheless, bacterioplankton growth and production were stimulated by carbon, nitrogen, and phosphorous additions, indicating that bacterioplankton biomass in this meso-eutrophic coastal lagoon is likely to be primarily bottom-up regulated. This study suggests that allochthonous carbon and inorganic nutrients via untreated sewage inputs or rainstorm runoffs into the lagoon have the potential to promote a quick response of bacterioplankton and, thus, increase the area and persistency of the “dead zones” within the lagoon. Our results reinforce the importance of an effective waste management plan for the area.

## Figures and Tables

**Table 1:** Results of one-way ANOVA performed to test the effect of different treatments (dark-control, light, and dark C + N + P) on bacterial abundance, biovolume, and biomass.

Parameter		DF^*∗*^	MS	*F*	*p*
Density (cells·L^−1^)	Treatment	2	0.4710	5.3	0.0154
	Error	18	0.0890		
Biovolume (*µ*m^3^)	Treatment	2	0.0031	0.3	0.7467
	Error	18	0.0105		
Biomass (mg·L^−1^)	Treatment	2	0.4734	4.6	0.0235
	Error	18	0.1017		

^*∗*^DF = degrees of freedom.

**Table 2:** Bacterial growth rates (hour^−1^ and day^−1^) and coefficient of determination (*R*^2^), production rates (hour^−1^ and day^−1^) and coefficient of determination (*R*^2^), and doubling time for light, dark-control, and dark C + N + P treatments. Bacterial growth and production rates were determined from the slope of the linear regression of ln-transformed bacterial abundance or biomass versus time (from time zero to the end of the exponential growth phase).

Treatment	Growth rate (h^−1^)	Growth rate (d^−1^)	*R* ^2^	Production rate (h^−1^)	Production rate (d^−1^)	*R* ^2^	Doubling time
Light	0.026^b^	0.626	0.86	0.018^b^	0.437	0.98	1.106
Dark-control	0.033^b^	0.789	0.74	0.021^a,b^	0.500	0.62	0.878
Dark C + N + P	0.056^a^	1.346	0.74	0.047^a^	1.128	0.67	0.515

Superscript letters indicate the result of ANCOVA and Tukey test for difference among slopes-*q* 0.05.

## Data Availability

The data used to support the findings of this study are available from the corresponding author upon request.
